# Parental Use of Social Media and the Internet in the Context of Their Child’s Genetic Neurodevelopmental Disorder: Mixed Methods Study Nested in the GenROC Cohort Study

**DOI:** 10.2196/76526

**Published:** 2025-10-14

**Authors:** Karen Jaqueline Low, Georgia Treneman-Evans, Sarah L Wynn, Jenny Ingram, John Powell

**Affiliations:** 1 Centre for Academic Child Health, Bristol Medical School, University of Bristol, Canynge Hall, Whatley Road, Bristol, BS8 3JZ, United Kingdom, 44 1173420000; 2 Department of Clinical Genetics and Genomics, University Hospitals Bristol and Weston NHS Trust, Bristol, United Kingdom; 3 Unique, Oxted, United Kingdom; 4 Nuffield Department of Primary Care Health Sciences, University of Oxford, Oxford, United Kingdom

**Keywords:** genetics, rare disease, social media, genetic neurodevelopmental disorders, parental support, mixed methods research, online health communities

## Abstract

**Background:**

Social media groups (SMGs) enable individuals with rare diseases to connect with one another and access instant support and advice. Accelerated diagnoses of genetic neurodevelopmental disorders (NDDs) over the last decade have driven rapid expansion of gene-specific SMG membership. Limited literature regarding parental use of SMGs in the context of managing their child’s NDD exists.

**Objective:**

The objective of our study was to determine and describe how parents use social media and the internet in the context of their child’s NDD.

**Methods:**

We undertook a mixed methods study of 477 parents within a cohort of children with NDDs (Genetic Rare Disease: Observational Cohort Study; GenROC). A total of 359 parents provided quantifiable survey responses regarding their use of social media. We also interviewed 17 parents to understand how they use SMGs and their views on the data held within these groups.

**Results:**

Our survey found 359/475 (75.7%) of parents use SMGs related to their child’s genetic disorder, and of these, 98.5% (354/359) are on Facebook. Most SMGs are closed, international, have more than 200 members, are specific to the NDD, and are associated with a corresponding charity or foundation. In total, 191/312 (61.2%) parents could not recall what they had consented to when joining the group with respect to the use of their posted data. Most parents trust the data (Likert scale 1‐10; mean 7.03, SD 1.98) that are shared but acknowledged the anecdotal nature of them. Parents found the most valuable element of the SMG to be shared lived experience with other families. Interview data from 17 parents were coded and analyzed thematically. A total of 4 main themes were identified: (1) SMGs for support and shared lived experience; (2) possible harms from participation in SMGs; (3) SMG composition, demographics, and dynamics; and (4) usefulness and use of data shared within the groups.

**Conclusions:**

This mixed methods study shows the evolving landscape of SMG use in neurodevelopmental disorders, highlights its benefits and downsides, and is widely applicable to all parent SMGs for specific niche medical conditions. Using the strength of these groups in a more collaborative approach in the future could prove useful to clinicians, families, and researchers alike.

## Introduction

Social media groups (SMGs) have dramatically changed the way individuals seek and share information online, particularly in the context of health care and are used by parents both as a support mechanism but also as a way of finding out information to help them look after their child [1,2]. Genetic neurodevelopmental disorders (NDDs) are rare with wide geographic spread, heightening the need for connection and increasing the information drought. Our recent literature review (Bogaert et al, unpublished data,December 2024) showed that SMGs empower parents by providing key information and a support role. Most parents use social media to help them manage their child’s NDD, and the majority use Facebook groups that are gene- or NDD-specific and are “closed” requiring authorized access by a group “admin” based on group-specific set criteria [3].

Regardless of the underlying disorder, parents use social media to acquire health-related information about specific medical concerns both before and after their child receives a medical diagnosis, and to seek out information based on other parents’ lived experience. This also provides a sense of support and community. Parents increasingly use this acquired information to discuss and sometimes challenge health care providers regarding the treatment options that are being offered for their child [4].

Medical health care was named as one of the key information needs identified in a study of parents of individuals with rare genetic disorders in a Facebook community group [5,6]. Tozzi et al [7] undertook a survey of 516 Italian families with rare genetic disorders to describe their internet use and found that 99% searched on disease characteristics, with 93% looking for therapies and 63% for alternative therapies, indicating high levels of need around treatments and therapy [7].

The number of diagnosed individuals with NDDs has changed substantially over the last decade largely due to changes in technology and access to genomic testing [8]. This means that the number of SMGs and number of individual members within each SMG have rapidly increased since 2010 and continue to climb [3,9].

However, the data about how SMGs help parents is limited, and with increasing numbers of diagnoses, the landscape of social media use is shifting. Our study aimed to answer the research question, “How do parents of children with NDDs use SMGs for support, and what is the perceived impact of these groups on their emotional well-being and access to clinical information?”

We aimed to describe this landscape and lived experience shared in SMGs by parents of children with NDDs in order to inform improved digital collaboration for the benefit of patients and families, clinical care, and scientific advancement.

## Methods

### Overview

We used a convergent mixed methods design for the surveys of parents and for the interviews with parents. This approach allowed for the simultaneous collection and analysis of qualitative and quantitative data, which were integrated during interpretation to provide a comprehensive understanding.

### Parent Surveys

This study is nested within the Genetic Rare Disease: Observational Cohort Study (GenROC) study, a UK-based cohort study of children aged 6 months to 16 years old with rare monogenic neurodevelopmental conditions (GenROC REC22/EM/0274) [10]. Parents were asked to complete 2 online separate patient questionnaires (PQs), PQ1 and PQ2 (completed between 2023 and 2025). Study data were collected and managed using REDCap (Research Electronic Data Capture; Vanderbilt University) electronic data capture tools hosted at the University of Bristol. REDCap is a secure, web-based software platform designed to support data capture for research studies, providing (1) an intuitive interface for validated data capture, (2) audit trails for tracking data manipulation and export procedures, (3) automated export procedures for seamless data downloads to common statistical packages, and (4) procedures for data integration and interoperability with external sources [11,12]. The parent questionnaires were developed with stakeholders (3 genetics researchers, 2 genetics charity representatives, 2 neurodisability pediatricians, 2 digital health academics, and 1 qualitative researcher) and patient participant involvement (PPI) input (6 parents of children with genetic NDDs and 1 adult with genetic NDD) and tested with 3 parents of children with NDDs using a think-aloud approach [13].

PQ1 and PQ2 both included social media questions (see Sections S5 and S6 in Multimedia Appendix 1) for the relevant sections of each survey, respectively, and development of PQ2 was informed by the results of the qualitative study described below.

Survey results were analyzed using descriptive statistics (in REDCap) for proportions and frequencies related to SMG use and parent attitudes.

### Parent Interviews

A total of 17 parents (5 fathers and 12 mothers) of 13 children were purposively sampled (Low et al [10], submitted) and interviewed using a topic guide which was informed by the literature, clinical expertise, and PPI input. Most parents were interviewed one-to-one but 4 couples chose to be interviewed together. In one case, a mother was interviewed with her child present throughout. Interviews took place between October 2023 and March 2024.

Interviews took place on Zoom (Zoom Communications) and were recorded, transcribed, and anonymized, then imported into NVIVO qualitative data management software (release 1.71; Lumivero) for coding and data management. All interviews were undertaken by the first author.

Data were analyzed following the principles of inductive thematic analysis [14-16]. An iterative approach was used to develop an initial codebook (deductive component) [17]. After 8 parents were interviewed, the process was paused to review the data and to develop and refine the coding matrix. This was then used for the remaining transcripts. We determined that we would continue with interviews until no new code categories were being generated and no new themes were created [15,16]. No repeat interviews were undertaken. The first 8 transcripts were coded by the first author, and additional codes that were not covered by the initial codebook were then added (inductive component). In addition, 20% of the transcripts were double coded by an experienced qualitative researcher who was not a clinician and had no previous experience of pediatric genetic disorders. All discrepancies were reconciled through consensus. Data are reported according to the Consolidated criteria for Reporting Qualitative studies (COREQ) guidelines [18].

### Data Integration

We integrated qualitative and quantitative findings during the interpretation phase using a side-by-side comparison approach [19].

### Ethical Considerations

The GenROC study received Research Ethics Committee approval on December 15, 2022, and Health Research Authority approval on February 9, 2023. All participants provided informed consent for the interview as per the GenROC study protocol. All information is deidentified to protect confidentiality and anonymity.

## Results

### Parent Surveys

A total of 474 parents completed the GenROC parent questionnaire. Of these, 97 (20.5%) said that they were not a member of an SMG for whom no further responses were collated. In addition, 359/474 parents (75.7%) stated that they were a member of an SMG. Multimedia Appendix 2 shows that 352/359 (98.5%) of the SMGs were on Facebook. Multimedia Appendix 2 shows the categories of genetic group, geographical distribution, numbers of SMGs used, and size of groups, respectively. The 4/359 (1.1%) participants who stated that the SMG was not on Facebook did not provide information regarding the location of the group. Only 1 parent answered that their child was a member of an SMG. Those who participated in more than 1 SMG did not consider the information to be conflicting.

Almost half (n=126, 44.8%) remembered being asked when joining to keep information within the SMG confidential, but almost as many parents (n=113, 40.2%) weren’t sure. Most parents (n=191, 61.2%) did not recall providing consent for their data from the SMG to be used elsewhere, with 30% (n=84) not being able to remember whether they had been asked. Of the 29 parents (10.3%) that recalled giving consent, 3 quarters remember giving consent to information being shared outside the SMG; 7 (24.1%) for any purpose; 15 (51.7%) to be shared with doctors and researchers working on the condition; and 7 (24.1%) could remember giving consent but could not remember any further details. A total of 65 (18%) respondents did not take part in any SMG with respect to their child’s NDD.

**Figure 1.**
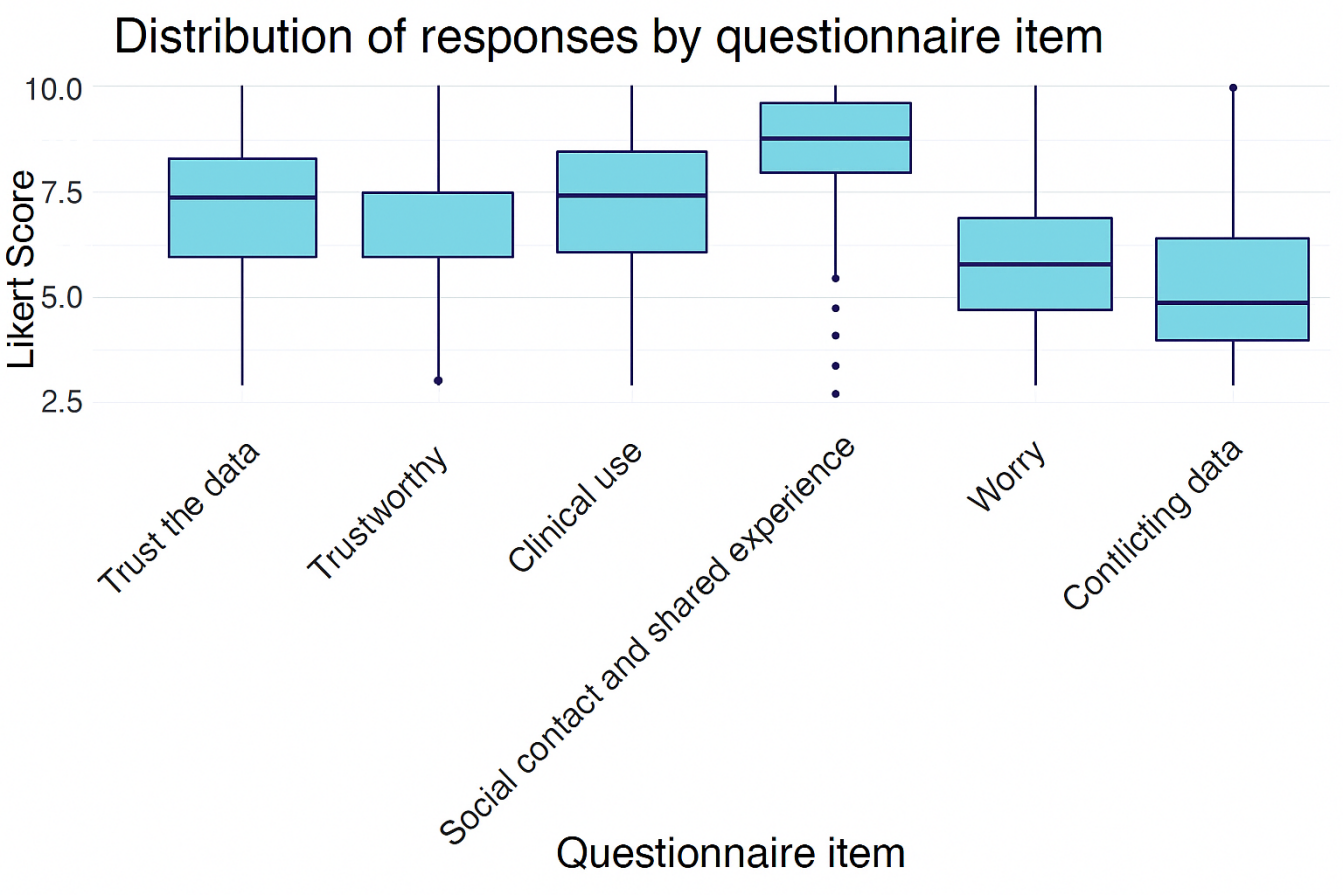
Distribution of responses by questionnaire item.

The results of the statements about attitudes toward the information held in the SMG are summarized in Table 1 (1 representing the lowest agreement and 10 the highest) with the distribution depicted in Figure 1.

**Table 1.** Responses by Likert scale to attitudinal statements.

Statement	Response rate^a^ (N=359), n (%)	Mean (SD)	Minimum^b^	Median (IQR)	Maximum^c^
Item 1: “I completely trust the information provided in the group.”	271 (74)	7.03 (1.98)	1	7 (5-8)	10
Item 2: “To what extent do you agree with the statement ’the information provided in the group is completely biased (for example a feature might look more common because people only respond to a poll if their child has the feature).”	263 (72)	5.77 (2.02)	1	5 (5-7)	10
Item 3: “To what extent do you believe that doctors or healthcare professions should be using the information from these groups when making clinical decisions?”	272 (75)	6.79 (2.03)	1	7 (5-8)	10
Item 4: “To what extent do you agree with the statement: The social media group helps by giving me contact with other people who have similar experiences.”	277 (77)	8.52 (1.85)	1	9 (8-10)	10
Item 5: “To what extent do you agree with the statement: I find that the information from the group can make me worried or anxious.”	269 (75)	4.69 (2.11)	1	5 (3-6)	10
Item 6: “I find that there is conflicting information in the different groups.”	145 (92)^d^	3.51 (2.2)	1	3 (2-5)	10

a Response rates were calculated as a percentage of parents who responded to the question out of a total count of 361, the number of parents who replied “yes” to being a member of a social media group (SMG) in the question already discussed.

b Lowest agreement.

c Highest agreement.

d n=158, as this question was only asked of people who had responded that they were part of more than one SMG with respect to their child’s genetic condition.

Most activity occurs in Facebook; 18 (8.3%) reported using a gene-specific WhatsApp group, with 5 (2.3%) using LinkedIn and 14 (6.4%) using Instagram. In addition, 10 (4.6%) follow an influencer on social media relevant to their child’s diagnosis: 8 followed a parent of a child with the same condition, 4 followed a parent of a child with another genetic condition, and 1 was a person with a genetic condition. Only 7 (3.2%) reported listening to relevant podcasts.

### Parent Interviews

**Figure 2.**
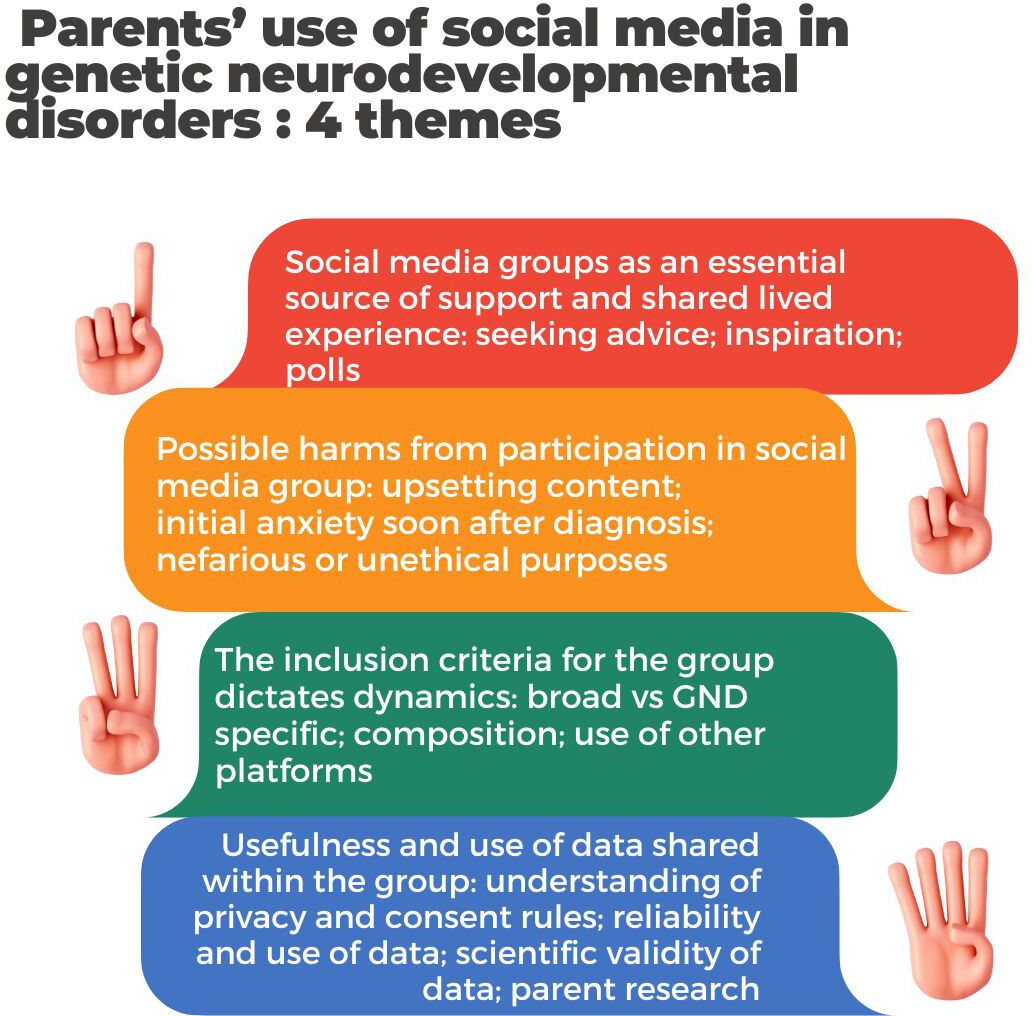
Summary of themes from parent interviews regarding parent use of social media groups (SMGs) with respect to their child’s neurodevelopmental disorder (NDD). GND: genetic neurodevelopmental disorder.

A total of 4 main themes (see Figure 2) were created from the parent interviews.

Quotes and participants are numbered in the tables and identified as mother or father.

#### Social Media Groups (SMGs) for Support and Shared Lived Experience

Table S1 in Multimedia Appendix 1 shows that some parents reported that clinicians signposted them to the gene-specific Facebook group after diagnosis as a possible avenue of support (Quote 1).

All parents described the SMG as providing them with a feeling of shared lived experience, which is vital for them as a coping mechanism (Quote 2).

The lack of natural history data in NDDs makes it hard for parents to know what the future might look like for their family, which they find very difficult. SMGs allow them to get more insight (Quote 3) and show how parents rely on the SMG as an easy way of accessing information about their child’s NDD and as a way of staying up to date and well informed (Quote 4).

Parents described some of the reasons for searching or posting. These included:

1. To help find information before attending an appointment with a health care professional for their child (Quote 5).
2. To get ideas for specific aids, interventions, days out, or toys that might be particularly appropriate or enjoyable for their child (Quote 6).
3. For medical advice about their child from other parents in the group (Quote 7).
4. For polls to find out about the prevalence of symptoms, features, and interventions, as well as to help researchers (Quote 8).

#### Potential Harms From Participation in SMGs

Table S2 in Multimedia Appendix 1 shows that parents described 3 subthemes of potential harms through participation in SMGs.

First, parents become upset by the content that they read or see in the groups.

Reading the posts in the SMGs can cause anxiety, and this can lead to reduced use (Quote 9) or have an impact on mental health (Quote 10).

Parents talked about how they are particularly distressed when they read posts about children in the group dying. A parent explained that it is not simply the subject matter itself but also the reflex anxiety it generates about one’s own child and a tendency to heightened anxiety and emotion within the group that can be difficult to deal with (Quote 11).

For some, the distressing content was about their own child. A parent explained that another member of the SMG was the first person to suggest the diagnosis for her child (Quote 12).

Second, joining a SMG soon after a child’s diagnosis can result in a spike in anxiety.

Participation in the SMGs can cause considerable anxiety in parents when joining soon after their child’s diagnosis (Quote 13). This was highlighted by a foster carer with her viewpoint on this initial spike of anxiety in biological parents (Quote 14).

The process of participating in the SMG, experiencing anxiety, and learning to manage that may be helpful in enabling parents to come to terms with their child’s NDD (Quote 15). Another parent described this phenomenon of initial anxiety and tried to highlight positive stories for newly joined families but recognizes that even she can get anxious in the groups (Quote 16).

Third, SMGs can be used for harmful purposes, but this is infrequent.

Parents described use of data in SMGs for unintended or unacceptable purposes, including a description of members of the group whose children had variants of uncertain significance that “didn’t look likely” and how they were trying to use information acquired in the group to try and obtain a diagnosis or “proof” for their child. A more extreme example of this misuse of data is where a parent describes a safeguarding case arising from a feeding tube Facebook group (Quote 17).

Parents reported that occasionally groups can be targeted for commercial purposes such as unsolicited marketing of equipment. While unwelcome, most of these instances are genuine; however, occasionally they can be preying on vulnerable families as part of a scam (Quote 18).

#### The Inclusion Criteria for the Group Dictates Dynamics

Table S3 in Multimedia Appendix 1 shows the inclusion criteria.

First, parent experience differs in groups that have broad inclusion versus specific criteria.

While most parents were members of NDD SMGs, some were members of broader SMGs as well. A foster parent reported preferring a broader fostering SMG to the NDD SMG as members were less likely to be offended by posts (Quote 19). In contrast, another parent explained that the mixed SMGs can result in a competitive dynamic between members who wish to be seen as the one having the most difficult time (Quote 20).

Second, group dynamics are impacted by geographical spread, size, and composition.

For some NDDs, the number of children being diagnosed has increased significantly, which has resulted in a much bigger SMG. This has resulted in some SMGs being split by composition (parents, grandparents, and researchers) and has impacted the content within the SMG (Quote 21). Some NDDs will have an international SMG as well as country-specific SMGs (such as UK families only). Parents explained the pros and cons of being part of one of the big international SMGs (Quote 22). Another problem with the international SMGs is that non-English speakers tend to receive fewer responses to their posts (Quote 23). As the SMGs get bigger and more children are diagnosed, more specific charities are set up, which can result in competition between charities within the groups. The same mother, who has set up an NDD charity, described some of the competing politics arising in a big SMG (Quote 24).

Third, group composition and dynamic may prompt the use of other platforms.

A parent explained that for the NDD, there is a UK-only Facebook group and then a bigger international one, hosted by a US charity. She reported that the UK SMG is very “quiet” and suspects that people think they have a higher chance of getting relevant information in the bigger SMG. However, there is still a desire for local or UK-specific support. For this reason, the UK-based charity is trialing a WhatsApp group just for UK families (Quote 25).

Another parent advocate who had set up a UK charity explained that she also uses LinkedIn for her charity work but in a professional capacity and in a different way from how she would use the Facebook group (Quote 26).

#### Usefulness and Use of Data Shared Within Groups

Table S4 in Multimedia Appendix 1 shows the usefulness and use of data shared within groups.

First, parent understanding about their data in the groups, privacy, and consent:

Parents varied in their recall of what they had been asked or what rules they agreed to when they signed up for the SMG (Quote 27). However, a parent was certain the only question they had been asked was whether their child had the specific genetic diagnosis.

Some parents considered the need to decide about sharing information in terms of their child’s best interests (Quote 28). Parents reported they have noticed that many parents in the SMGs appear to trust it implicitly and will share highly confidential data (Quote 29). However, some parents stated that they had little confidence in anything they shared being kept private (Quote 30) and that there are no guarantees that the people who join are genuine (Quote 31).

Foster carers described a nuanced aspect regarding the privacy of data and the need to protect a looked-after child’s identity, particularly within a small SMG in which a birth parent may be a member if they have the same genetic condition as the child (Quote 32). This need for heightened privacy limits their ability to access the same amount of support from the SMG as others.

Second, parent views on the reliability and use for the data in the groups:

Parents discussed how they assess digital content for scientific validity. All report using search engines, mostly Google, as a starting point. Many reported that they would trust information that was in a scientific journal or if it was affiliated with a university in some way (Quote 33).

Third, parent views on the trustworthiness of the data in the groups:

Parents perceived a bias in the frequency of posters, but there were differing views. A parent commented that “you do tend to hear more from the people that are having the worst time”(#15, father), whereas another parent reported that the parents who post most have the “mildest” children (Quote 34).

Nonetheless, parents feel they are objective about the information being presented and consider it useful but anecdotal (Quote 35). Another parent talked about the need to verify and fact-check information from within the SMGs (Quote 36).

Fourth, parent-independent research and participation in research:

Although a lot of data are being collected in SMGs, most parents doubted that useful information could be extracted, or that it would be legal to do so, but felt that it was helpful to be able to search old threads such as regarding treatment options (Quote 37). Parents suggested that the data could be used to identify themes that could guide researchers toward avenues for future studies (Quote 38).

A parent reported that a large language model application is being used within their group that she found out about recently at the family conference (Quote 39).

Most parents described researching their child’s specific genetic variant themselves. For some, this would be through the SMG, and some parents would develop expertise and answer other members’ questions about the variants within the group. To gain understanding and expertise, they use online data sources with a search engine as a starting point (Quote 40).

Many parents reported taking part in online registries and in rare disease portals where they are asked to complete questionnaires at intervals. Parents considered this as being very different from their posting in Facebook groups, thinking of the former as scientific research and the latter as anecdotal and social.

### Integration

Table 2 shows the side-by-side comparison approach of the qualitative and quantitative results. Mapping of the themes to the survey findings confirmed that parents highly valued SMGs for emotional support and shared experiences, though some reported moderate concerns about anxiety. Group composition influenced perceived support. While parents generally trusted SMG data, they acknowledged potential bias and privacy concerns.

**Table 2.** Joint display table mapping qualitative themes to quantitative results.

Qualitative theme	Subthemes	Mapped quantitative findings	Interpretation
Support and shared experience	Signposting by professionals, emotional connection, and practical advice.	High agreement (8.52/10) on SMGs^a^ providing contact with similar experiences.	SMGs are a vital support system for parents.
Potential harms	Anxiety, distressing content, and misuse of data.	Moderate agreement (4.69/10) on SMGs causing anxiety.	Harms are present but not dominant; qualitative data adds nuance.
Group composition and dynamics	Inclusion criteria, international versus local, and platform choice.	A total of 98% (354/359) of SMGs on Facebook; some use WhatsApp or LinkedIn.	Group structure affects experience; larger groups may dilute support.
Usefulness and data trust	Privacy concerns, trust, bias, and research use.	Mixed scores: trust (7.03), bias (5.77), and doctor use (6.79).	Parents value SMG data but recognize limitations and risks.
Independent research and advocacy	Self-directed learning, registry participation, and data mining.	Not directly surveyed.	Parents are proactive in research; SMGs serve as informal knowledge hubs.

a SMG: social media group.

## Discussion

### Principal Findings

Use of SMGs is highly prevalent in this cohort and provides support and information to fill an existing gap. However, the information in these SMGs is largely anecdotal, requires a degree of critical assessment, and can cause anxiety. Understanding SMG use and activity is important to ensure optimal support for parents, to address information needs adequately, and to improve collaboration between parents, clinicians, and researchers.

Some parents told us in interviews that they were directed to the SMG by their child’s health care professional who suggested it to them as an avenue of support and information. Unfortunately, this was not specifically quantified within our parent survey but is consistent with a previous study in which a quarter of parents were directed to the group by their child’s health care professional [4].

Parents value these SMGs very highly as shown by their high Likert response and qualitative analysis that is consistent with the literature [5-7,9,20,21]. Most parents view the SMGs as an opportunity to connect with other families who are in the same situation and develop real connections with them and friendships. Parents particularly valued the chance to understand the spectrum of the NDD by looking at other members’ posts about their children. Having members with older or adult children allowed them to get some understanding about the long-term outlook for their child, which is consistent with previous literature [5,21-23].

Some parents also use the SMG to obtain quasi-medical advice from other members and would trust it implicitly. We cited an example where a parent chose not to be present for an acute review of her child based on medical advice provided within the group. An important factor here may be responsiveness—Facebook groups provide rapid responses and ongoing support and feedback [24], which may empower a parent to make a decision to “watch and wait.” However, some parents in our study reported concerns about using the group in this way and expressed concerns that the level of trust placed in the reliability of such advice might be unfounded or too high. This is echoed in a previous analysis of Hirschsprung disease in which the Facebook group was shown to have reach and responsiveness but lacked consistent evidence-based guidance in the posts [24]. A particular use detected in our study was to prepare a parent before a child’s health appointment to ensure they knew what to discuss and request from the provider. While this behavior is cited as common among parents of all children [1], it is more nuanced in NDDs where parents may feel that they need to undertake this preparatory research to negate their clinician’s lack of expertise. If clinicians recognize this phenomenon is occurring, they can use this as a route to provide better collaborative support for parents. An example of this might be suggesting to parents that they may read information in SMGs that they might want to note down and come prepared with some questions or even inviting parents to send questions in advance, which might allow the clinician adequate time to do their own research. A real-world scenario could involve a clinician opening with the question, “Have you read anything on the Facebook group that you want to discuss with me today?”

Most parents indicated high levels of trust in the SMG (by the Likert scale responses) and are not worried about their data or put off posting for this reason, although a small proportion did discuss their concerns around this. A study in 2022 of 231 parents indicated that parents do express concern about privacy issues and that the SMG’s privacy setting can be a critical factor influencing active support group participation [4]. However, almost half of our respondents could not recall whether they had been asked to agree to any statements regarding consent or privacy when joining, but continued to participate in the SMGs. The analysis of parent interviews suggests that parents with the highest levels of concerns around privacy would be most cautious about what they post in the SMGs. It is also noteworthy that an SMG appears to be using some form of natural language processing model to mine the data for themes. This raises questions regarding consent within the groups and use of data.

Looked-after children represent a particularly vulnerable group within social media, and foster or adoptive parents are required to hide their identities to protect them. This can be difficult with a rare NDD where patient numbers may be very small. Genetic variants can be unique to a family or individual and could theoretically be identifiable, and so this may limit the foster or adoptive parents’ options for interactions and questions within an SMG, limiting their support opportunities compared to other families.

SMG participation is not without its downsides. A spike in anxiety can occur initially after joining, particularly when this occurs soon after receiving a diagnosis (which is the most common time that parents join). Nonetheless, most of the survey respondents did not agree that the SMG made them worry more. The potential negative impact is sparsely discussed in the literature to date [6].

There was recognition of posting bias within the SMGs, but some of the parents who talked about this felt that they were able to assess the posts and come to conclusions about bias themselves. However, parents trust the data that are shared, as evidenced by the high levels of trust reported by the Likert scale, and parents report making health care decisions about a child presenting with new symptoms based on the SMG responses.

Our results show that there are many NDDs that have more than 1 SMG, which families can join, consistent with a previous estimate that 33% of monogenic disorders had more than 1 SMG [9]. It is likely that this percentage will have increased based on the rapid acceleration of diagnosis in these conditions. The same study found that SMGs with large numbers of members were more likely to be active and have the most posts [4]. This is in keeping with our findings that parents are likely to go to larger SMGs where they feel they have the highest chance of getting data that they seek. However, these larger SMGs appear to have downsides, as does the presence of more than 1 group per NDD. Our results suggest that there are increasing politics and tensions within groups, particularly where there is a suggestion of increasing competition for members within NDD charities and foundations, and that the SMGs may be being used to steer members toward particular organizations. These changing dynamics are scarcely explored in the literature to date, which may be because this is a newly evolving phenomenon with time and rapidly increasing numbers of diagnosed conditions.

The changing dynamics within groups have resulted in some groups trying options that are not Facebook. This has included WhatsApp as an option for 1 UK-based group who has fewer than 50 families. The decision was made to try and create a more UK-specific option for families. However, functionality may become a barrier as the group increases in size, as might its lack of interactive options and easy search tools. Our results otherwise suggest that parents are not using other platforms very much for this specific purpose. A smaller number of parents use LinkedIn, but particularly for professional advocacy purposes.

Facebook groups are closed, and it appears this is vital for ensuring families feel able to participate in a way that protects their privacy needs [8]. Nonetheless, our findings suggest that SMGs could increasingly be used by researchers to engage with patient groups better. In addition, we report several examples within groups to determine appetite for a topic of research or assess the availability of data before embarking on a research proposal. Using poll results could also identify important topics that matter to families. Researchers need to establish positive connections with group administrators to do this successfully. Very little exists in the literature to date on this theme, again reflecting this rapidly advancing field and area.

### Strengths and Limitations

This study uses a large cohort of parents of children with a mix of genetic conditions from across the United Kingdom. The majority of survey respondents and interviewees were mothers, but some fathers were included. Our methods were able to capture responses from parents who do not use SMGs or who have stopped doing so, as well as regular users. The lead investigator is a clinical geneticist, but the second coder for the interviews is a nonclinical experienced behavioral and psychological qualitative researcher. Parents described negative experiences with health care professionals suggesting that they felt able to express their views despite the researcher’s clinical background. The PQ2 survey had questions that had hoped to capture parental behavior change regarding SMG with time but were limited in the short interval between survey administrations for some families who were recruited later.

### Conclusions and Implications for Practice

Data poverty in rare diseases drives parents to do their own research and propels them into SMGs for connection. Parent-reported data in groups reflect lived experience data. SMGs are essential for parents as a space to connect and compare experiences and may represent an opportunity as an avenue to drive forward research connections and priorities and allow for opportunities for advocates and peer mentoring to flourish. Parents use SMGs as a quasi-clinical advisor in some situations and for preparation before attending health care appointments—this could represent a way in for enhanced collaboration with parents in the clinic. The rapidly changing landscape of NDDs may shift the dynamics within groups that could result in an evolution in the number of groups and platforms used. Limited reports exist on the negative aspects, and these appear to be a minority, but some parents reported reducing or stopping their use of the group altogether, which may be associated with these problems. Further research should ascertain if this is an increasing trend, as this could result in a support gap for parents in the future. Changing behavior over time and evolving information needs represent an avenue for future research.

## Supplementary material

10.2196/76526 Multimedia Appendix 1 Quotes from parents for themes 1-4 and the list of Genetic Rare Disease: Observational Cohort Study (GenROC) consortium members.

10.2196/76526 Multimedia Appendix 2 Demographics of social media group (SMG) use.

## Abbreviations

NDD: neurodevelopmental disorder
PPI: patient participant involvement
PQ: Parent Questionnaire
REDCap: Research Electronic Data Capture
SMG: social media group
