# Supplementary material for: Parental Use of Social Media and the Internet in the Context of Their Child’s Genetic Neurodevelopmental Disorder: Mixed Methods Study Nested in the GenROC Cohort Study
Source: JMIR Pediatr Parent. 2025 Oct 14;8:e76526. doi: 10.2196/76526 (PMC12520642; doi:10.2196/76526)
Supplement: Multimedia Appendix 1 [file pediatrics-v8-e76526-s001.pdf]

Table S1. Quotes from parents- SMGs for support and shared lived experience

Theme	Quote number	Quote	Source
**Signposting by professionals**	1	"So then I was directed to the Facebook group, which is really the only support group, and to contact the Office for Rare Conditions which is the Scottish version of Unique, and then... and Unique, and I was told to give them a shout and see if I could [[get] any help."	#16,mother
	2	"...but as parents it's scary for us because we don't actually know what's going on with our son...But when we listen to other parents on the group we know kids are different but they're all going through exactly the same thing, and the same journey."	#1,mother
**See the future**	3	"We can see what a day in the life for their family [...] going back to the progression and what the future could possibly hold for [son], [...] and you get the camaraderie of being part of a group that can work together."	#6,mother
**Stay informed**	4	"You just find so much more out from other parents, and then with the [X] group they post all the research and everything in there, [...] and they post the conferences. So it's an easy way to get to all of the information."	#3,mother
**Reasons for posting (preparing for appointment)**	5	"So I do go onto the Facebook group but I typically go on to look for something ahead of an appointment. [...]. So I looked up the information that was on there, [...] [...] because she's got a squint, and I wanted to know if I could get the consultant to look at her eyes to check for those optic nerves."	#16,mother
**Reasons for posting (to get ideas)**	6	"But if they're like have you tried sensory play in a dark room? Or have you tried taking him to an aquarium or something like that? We're like oh okay that really helps...Or suggestions of toys ...[...] like birthdays when family and friends want to buy things,	#6,mother
**Reasons for posting (medical advice)**	7	"I said, "He's starting to get a lip drop..." [...] another parent said, "Oh actually my child had, when they had a virus it went into[...] and I said, "Okay, he has just had a virus, okay I don't need to worry," I don't need to now take him to hospital ....."	#5,mother
**Reasons to post (polls)**	8	"I do polls actually, we get really good information, but I always tell people what it's for, ... so for example I think [researcher] is hopefully going to do an MRI study, so he needed to know how many patients actually have more than one MRI[...]so I could say yes actually we do have enough, and how many would be happy to actually be part of a study."	#10,mother

This is a Multimedia Appendix to a full manuscript published in the J Med Internet Res. For full copyright and citation information see http://dx.doi.org/10.2196/jmir.76526

Table S2. Quotes from parents -Possible harms from participation in SMGs.

Theme	Number	Quote	Source
Content increases worry about what might happen	9	"So I don't want to start researching [...] and then thinking the worst and then he doesn't have that[...] I feel like I don't want to waste the energy doing all of that, and worrying, and then stressing over him."	#4,mother
Impact on own mental health	10	"It's not enjoyable to read articles or watch videos about children suffering, or having 50 plus seizures a day [...] even if you know that's not necessarily you. It's quite a negative thing to be constantly seeing [...] it can weigh quite heavy on your mind and your emotions "	#6,mother
Child death	11	"When we have a death there's suddenly all this drama[...] you suddenly get all the other parents going oh my god is my child going to die? [...] But yeah, I think you get more aware of the fatalities, which probably isn't very good for anybody's mental health, and they get shared around a lot, so that's pretty depressing."	#10,mother
Distress caused by interactions with others in the group	12	"They said she looks like she has [X]..I was insulted by yeah, because I'd read it and was like no, I'm sure she's just got [x], even though they told me [my child] was small. [...]. It's like telling somebody you might have cancer but you might not[...] the person would probably [...] prefer to be told nothing until they knew I would think."	#10,mother
Joining soon after diagnosis spikes anxiety	13	"I think it did to begin with, because I thought is she going to be like that? Or how it was already hard how she is now, let alone how she could be, and I think it was the uncertainty..."	#14,mother
Looked after perspective	14	"... because I don't have those genetic connections to these children[...]I haven't passed this to them... So people [...]get upset because obviously they're going is it me?...They're coming to terms with what they thought might have been a short term issue, actually becomes a lifelong issue, because actually if it's a genetic problem it ain't going to go away."	#17,mother
Dealing with anxiety may help comE to terms with diagnosis	15	"You tend to hear from the people that are having the worst time. [...]you can see some things that make you anxious, but you're going to be anxious anyway...learning how to deal with that anxiety is the journey that you're on anyway, so it's like a necessary downside."	#15,father
Other parents support newly diagnosed families	16	"some families have commented on the Facebook forum that they have just been diagnosed and it's been really overwhelming and upsetting, "Is there anything positive to hear? I'm so terrified," [...]actually there is lots of positive stuff on there, [...] we will always post on there and reassure them that this is a safe place, and that's why you'll see the overwhelming stuff, because this is the place where we can share that, that absolutely there's lots of positive stuff as well..."	#11,mother
Groups can be used for unsavoury purposes<br>-Safeguarding	17	"There was a mother asking for help, and I described everything about when [child] got his feeding tube, and the fights with gastro to get the fund, going to get his button put in and everything, [...] and it turned out she was abusing her child. He didn't need a feeding tube, and she was using our group to get the information to tell the doctors. It went to court, and they used my words as part of the evidence in court to prove that she'd been going digging for information."	#3,mother
Groups can be used for unsavoury purposes<br>-Scams	18	"I know that on the special needs websites for equipment that people try and scam you, trying to purchase for example a specialist wheelchair and whatnot, and marketplace and whatnot, and people are trying to purchase equipment that is really expensive at the normal price."	#6,mother

Table S3. Quotes from parents -Group composition and dynamics

Theme	Number	Quote	Source
**Broad vs Specific**	19	"(I prefer the) Fostering groups, because nobody is hurting anybody else's feelings, nobody is very fragile, and you can just say it how it is."	#17,mother
**Prefers GND specific**	20	"you end up feeling a bit like the oppression Olympics where whose kids are the worst off, and I can't complain that mine isn't verbal, because other people's children will not only be non-verbal but will never walk, [...]. you end up starting to feel quite othered in a group that's supposed to be inclusive"	#16,mother
**Subgroups**	21	"There's a private group just for parents and carers[...]and then there's a public forum, so the grandparents have joined that one as well... But definitely the theme of the stuff [...] is much more people introducing themselves and saying they've just had this diagnosis[...] a lot more of that [...] in the last year than there was at the beginning."	#12,mother
**International vs country specific**	22	"For certain treatments it's useful. [...]. But also because it's a much bigger group it's quite chaotic, and you just... there's just loads of posts from people just having a terrible time kind of thing. Yeah, but because their health system is so different it doesn't seem so useful."	#15,father
**First language**	23	"It's getting a bit too big now, because obviously more people are getting diagnosed, and I think what I see happening is that some of the people whose English isn't a first language their questions get ignored. [...]. I always try to answer theirs, because I feel like they're the ones that need the most support, they don't have any community."	#10,mother
**Group politics**	24	"The newest [country] one is not supportive of our charity, which I don't understand, because obviously we're not competing...the Facebook group is more I don't know if political is the right angle, but they use it definitely for their own means as well as for support. [...]. So they friend them immediately[...], and they basically are accumulating families for the knowledge of their charity through this Facebook group."	#10,mother
**Group composition/dynamic can prompt use of other platforms**	25	"The UK group just doesn't get enough traffic...Because it was feeling like it was just disappearing, and obviously new parents want this community really. So we came up with the idea only to have a WhatsApp group, because your counts were so small. Obviously if you have a few hundred people in a group it's not going to be possible..."	#10,mother
**Use of LinkedIn for professional purposes**	26	"So that's how I've reached out to all of the doctors, and the scientists and the researchers. The more I post about a certain topic the more I see people that are involved in those topics. It's how I become aware of conferences and congresses, and it's a really quick informal way of reaching out to those to be, "Hey we're here, can we come and present at your conference?" And, "Is anyone interested in this condition?"	#11,mother

Table S4. Quotes from parents -Usefulness and use of data shared within SMGs

Theme	Number	Quote	Source
Data privacy, consent	27	“I can’t remember what they are to be honest. I feel like one of them was probably about confidentiality, about it is a closed group, you have to ask to join, you do have to be a carer…”	#5,mother
Concerns about child’s right to privacy	28	“I think with her consent is always going to be difficult because she won’t understand. So I think it’s always down to me and her dad to decide what’s best for her at that time, so if at 16 we think actually this is enough now, there’s no need to do this, we have information that we have that’s relevant for her then we won’t do it, but we will always do it in her interest, not for us but for her.”	#14,mother
High levels of trust in the group	29	“But it’s definitely people share everything on it, it’s not secure. People are a bit silly, and will give genetic report to anybody, like to any other members,.”	#10,mother
No trust	30	“My assumption is that by posting I’ve just given them open access basically to anything that I post. […] I wouldn’t post anything that I didn’t want shared.”	#3,mother
	31	“It can be genuine people, it can be people who are not genuine. So normally you have to say what your connection is and why you want to join, you can make that up… that’s what people have to remember, that actually it’s social media, and there are no givens, there’s nothing that’s concrete on there.”	#17,mother
Privacy and looked after children	32	“So as foster carers you can’t share photos of your children on social media, you can’t really share any identifiable information, […]So I just call myself foster mum, and I have a picture of a teddy bear, [..] I don’t think his birth family will be scrolling through the internet trying to find him, but […] you have to just cover yourself for these things, yeah, which can be frustrating.”	#5,mother
How parents evaluate data	33	“Just if it was a scientific paper. […] if something turned up on PubMed with an abstract and it made sense then I’d take that as being pretty reliable, […]… some of them are just looking at 12 kids or whatever, and they’re all quite small studies. But yeah, definitely placing any published paper above random opinions, but not really going any deeper than that I suppose.”	#15,father
Perception of bias	34	“So the parents who are most active seem to have milder children, like the ones that are walking, and that should be a minority based on the data, and I just wonder if […]maybe the families with children who are really struggling aren’t sat on social media.”	#10,mother
Data is anecdotal	35	“ I’m lucky in that my husband and I we both study sciences at university, so we both have a very good understanding of what is a good level of evidence. […] people will give you their experience as if it were the gospel truth, because obviously it is to them. But it doesn’t mean that’s what’s going to happen to my child”	#16,mother
Need for fact checking	36	“So you get people going on about it a lot, saying, “This girl’s epilepsy was completely cured by cannabis,” and it wasn’t quite like that. It helped her a lot, it didn’t cure her… […], I guess I’m just confident to navigate these things, and yeah look at the actual research and stuff.”	#15,father
Ability to search on threads	37	“But could you do anything with it? […] some of it’s very personal as well. […]. So every six months somebody is asking about [treatment] or something, and then whoever’s around will give their opinions, and they could go back and look at what people have said before. […]Yeah, I guess there would be so many privacy problems trying to extract anything from it, that you probably can’t.”	#15,father
Data could guide themes for research	38	“But it’s just poorly structured research basically. […] if you’re looking for high quality research you would do it in a better way. […]You can perhaps get some insight, and some points of what to look into further from looking at the group…”	#7, father
Covert research in group	39	“We also have this clever […] data collection something or other attached to it, which doesn’t take any personal information at all, but it syphons out frequently mentioned or referred to topics, so that it can pick up more on the anecdotal family feedback as opposed to what the researchers decide to talk about.”	#11,mother
Self-directed research	40	“I think if you type the actual variant code into Google […] I found a university in China had an [gene name] database online which tracked all the different mutations, and all the different papers that had mentioned each mutation and stuff. So I did a lot of research around there, but that didn’t really make much difference to anything except I learnt a lot about genetics I suppose.”	#15, father

S5. GenROC Social media Parent questionnaire – PQ1

In this section we will ask you to tell us about your social media/internet use related to your child's genetic condition. This information is really helpful given this is one of our particular areas of focus. Thank you.

Are you (or someone in the family) a member of a social media group that is specific to your child's genetic condition?

This could include a gene specific whatsapp group, facebook group, or other social media site or app.

Please select all options that apply.

- ☐ No
- ☐ Yes, I am
- ☐ Yes, my child is
- ☐ Yes, another member of my family is
- ☐ I don't know the answer for family members

With respect to your own social media use: Are you a member of more than one social media group relating to your child's genetic condition?

- ○ Yes
- ○ No

Is the social media group on facebook?

(If you use more than one group please answer about the group that you use the most)

- ○ Yes
- ○ No

In which social media site/webpage/app is the group held?

____

Is the group specific to:

- ○ the gene?
- ○ A group of genes?
- ○ A group of conditions?

In terms of the people who are members of the group are people mostly from

- ○ The UK only
- ○ The USA
- ○ Its a mix from lots of different countries
- ○ I am not sure

[optional] how many members are in the group?

- ○ 0-10
- ○ 10-20
- ○ 20-50
- ○ 50-100
- ○ 100-200
- ○ 200+

Is the social media group associated with an official patient support organisation or foundation specific to the genetic condition?

- ○ Yes
- ○ No

When you joined the group originally were you asked to agree to keep information within the group confidential?

- ○ Yes
- ○ No
- ○ Don't know

Have you been asked to provide any sort of consent for the information you provide in the group to be used elsewhere (eg for research or clinical use)

- ○ Yes
- ○ No
- ○ Don't know

Regarding the consent that you gave please select all that apply:

- ☐ I remember giving consent for information to be shared outside the group for any purpose
- ☐ I remember giving consent for information to be shared outside the group with researchers and doctors working on the condition
- ☐ I remember giving consent but cannot remember an of the details
- ☐ other

Please provide more information about this

____

To what extent do you agree with the statement 'I completely trust the information provided in the group '

Don't trust at all — Neutral — Trust completely

(Place a mark on the scale above)

To what extent do you agree with the statement 'the information provided in the group is completely biased (for example a feature might look more common because people only respond to a poll if their child has the feature)

Completely biased — Neutral — Not at all biased

(Place a mark on the scale above)

To what extent do you believe that doctors or healthcare professions should be using the information from these groups when making clinical decisions?

They shouldn't use it at all — Neutral — Yes they definitely should

(Place a mark on the scale above)

To what extent do you agree with the statement:
The social media group helps by giving me contact with other people who have similar experiences

Not at all — Neutral — Yes, completely

(Place a mark on the scale above)

To what extent do you agree with the statement:
I find that the information from the group can make me worried or anxious

Never — Some of the time — Almost every time I go on the group

(Place a mark on the scale above)

You said that you use more than one social media group to help you with your child's genetic condition. How many groups are you a part of?

- ○ 2
- ○ 3
- ○ 4
- ○ 5
- ○ 6
- ○ 7
- ○ 8
- ○ 9
- ○ 10 or more

To what extent do you agree with the statement:
I find that there is conflicting information in the different groups

Not at all — Neutral — Yes, completely

(Place a mark on the scale above)

S6. GenROC Social media Parent questionnaire – PQ2

You may remember we asked you some questions in the last questionnaire about how you use social media to help you as a parent of a child with a genetic condition. This next section asks some other related questions that will help us understand this better.

Previously we asked you about your use of social media relating to your child's genetic condition.<br><br>Since you completed the last GenROC questionnaire select ALL that apply:	☐ I have started using the Facebook group<br>☐ I have stopped using the Facebook group<br>☐ I have reduced my use of the Facebook group<br>☐ I have joined a new Facebook group<br>☐ I am using a gene specific WhatsApp group<br>☐ I am using LinkedIn<br>☐ I am using Instagram.<br>☐ I follow an influencer on social media (related to the genetic condition)<br>☐ I listen to podcasts related to my child's condition/genetics<br>☐ Nothing has changed<br>☐ Other
Is the influencer an:	☐ A Parent of a child with the same condition as yours<br>☐ A parent of a child with another genetic condition<br>☐ A researcher<br>☐ A person with a genetic condition<br>☐ Other
You said 'other' - can you explain more?	______________________________

S7. GenROC Consortium Members

Suzanne Alsters
Ruth Armstrong
Tazeen Ashraf
Queenstone Baker
Meena Balasubramanian
Diana Baralle
Jonathan Berg
Marta Bertoli
Ishita Bhatnagar
Thomas Boddington
Moira Blyth
Catherine Breen
Helen Brittain
Lisa Bryson
Jenny Carmichael
Emma Clement
Tessa Coupar
Anna de Burca
Cristina Dias
Abhijit Dixit
Alan Donaldson
Andrew Douglas
Jacqueline Eason
Sahar Elkady
Nour Elkhateeb
Fayadh Fauzi
Elaine Fletcher
Helen V. Firth
Nicola Foulds
Caroline Furnell
Andrew E. Fry
Laura Furness
Jennifer Gardner
Gabriella Gazdagh
Merrie Gowie
Abigail Green
Asma Hamad
Rachel Harrison
Verity Hartill
Lizzie Harris
Eleanor Hay
Jonathon Hoffman
Jenny Higgs
Simon Holden
Daniela Iancu
Rachel Irving
Vani Jain
Rosalyn Jewell
Diana Johnson
Gabriela Jones
Beckie Kaemba
Arveen Kamath
Ayse Nur Kavasoglu
Tabassum Khan
Mira Kharbanda
Sophie King
Usha Kini
Alison Kraus
Ajith Kumar
Katherine Lachlan
Neeta Lakhani
Wayne Lam
Anne Lampe
Abigail Lazenbury
Harry Leitch
Helen Leveret
Samuel Liebert
Jessica Maiden
Anirban Majumdar
Alison Male
Alisdair McNeil
Ruth McGowan
Holly McHale
Catherine McWilliam
Jonathan Memish
Lara Menzies
Radwa Mohamed
Tara Montgomery
Oliver Murch
Fiona Osborne
Michael Parker
Caroline Pottinger
Vijayalakshmi Ramakumaran
Thiloka Ratnaike
Ruth Richardson
Lisa Robertson
Alison Ross
Claire Searle
Wofah Selah
Resifina Seyara
Charles Shaw-Smith
Suresh Somarathi
Charlotte Stanley
Edward Steel
Helen Stewart
Kerra Templeton
Riya Tharakan
Madeline Tooley
Fleur S. Van Dijk
Mohamed Wafik
Emma Wakeling
Elizabeth Wall
Amy Watford
Patricia Wells
Louise Wilson
Emily Woods
