# Supplementary figures and images for: Parental Use of Social Media and the Internet in the Context of Their Child’s Genetic Neurodevelopmental Disorder: Mixed Methods Study Nested in the GenROC Cohort Study
Source: JMIR Pediatr Parent. 2025 Oct 14;8:e76526. doi: 10.2196/76526 (PMC12520642; doi:10.2196/76526)

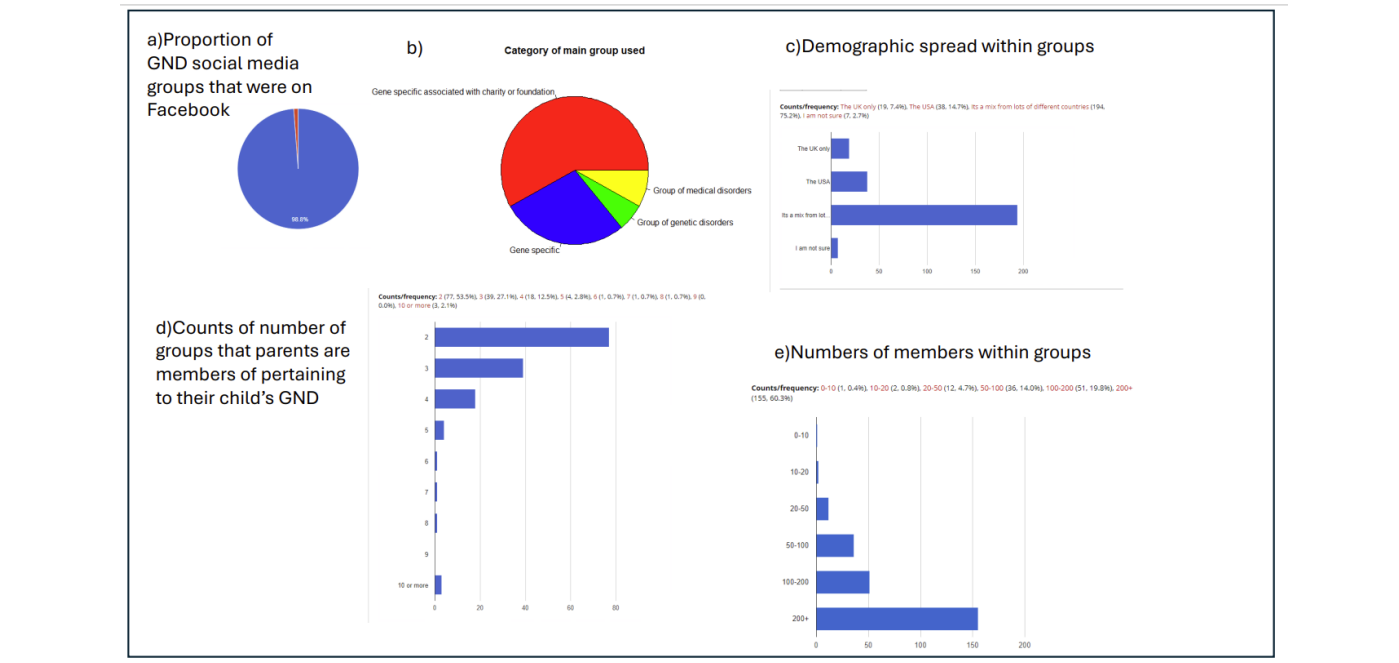

Supplement: Multimedia Appendix 2 [file pediatrics-v8-e76526-s002.png]
